# Group B *Streptococcus* Sepsis with Cardiac Abscesses in a Neonate with Migrated Umbilical Catheter: Literature Review

**DOI:** 10.1055/a-2770-5011

**Published:** 2026-01-15

**Authors:** Trenton Judd, Mimily Harsono, Jie Zhang, Fatima Mir, Massroor Pourcyrous

**Affiliations:** 1 Division of Neonatal-Perinatal Medicine, Department of Pediatrics, University of Tennessee Health Science Center, Regional One Health Hospital NICU, LeBonheur Children's Hospital NICU, Memphis, Tennessee, United States; 2 Division of Pediatric Pathology, Department of Pathology, University of Tennessee Health Science Center, LeBonheur Children's Hospital, Memphis, Tennessee, United States; 3 Department of Pathology, University of Tennessee Health Science Center, Regional One Health Hospital, Memphis, Tennessee, United States

**Keywords:** cardiac abscess, extreme premature, extreme low birth weight, group B *Streptococcus*, myocardial abscess, neonate, umbilical catheter, umbilical artery catheter, umbilical venous catheter

## Abstract

Group B *Streptococcus* (GBS) is the leading cause of sepsis, pneumonia, and/or meningitis in neonates. Insertion of an umbilical catheter (UC) is a common practice in neonatal intensive care for primary central vascular access in extreme premature neonates. UC is used for the administration of intravenous medications, parenteral nutrition, blood samplings, and continuous central blood pressure monitoring. Malposition or migration of UC tends to occur in extreme premature infants with risks of multiple complications. We present a case of an extreme premature neonate who developed fatal GBS sepsis with autopsy findings of multiple cardiac abscesses in the myocardium but not in any other organ. GBS sepsis with isolated multifocal myocardial abscesses leading to sudden fatal clinical deterioration has not been described previously. In this review, we describe the plausible pathological mechanism of this rare presentation. Intracardiac migrated UC, in conjunction with rhythmic heart contractions and intracardiac blood flow dynamics, can cause direct trauma to the endocardium. Damaged endocardium can be a potential nidus for bacterial overgrowth and abscess formation that ultimately may lead to cardiac failure. Therefore, the correct aseptic technique of securing and management of UC, and daily assessment of UC position are recommended to prevent complications associated with catheter migration.

## Introduction

Group B *Streptococcus* (GBS) or *Streptococcus agalactiae* is a common gastrointestinal and genitourinary bacteria that is often described as commensal in healthy adults. However, for individuals with low immune system status, particularly neonates, GBS infection is the most common cause of pneumonia, sepsis, meningitis, or invasive bacterial infection, with an estimated incidence of 0.2 to 0.4 in 1,000 live births, while extreme premature neonates (gestational age [GA] <29 weeks) or extreme low birth weight neonates (birth weight <1,000 g) are at much higher risk of infection or mortality. 1 2 GBS infection is classified as (1) early-onset (EO) that presents at 0 to 6 days of life with an estimated incidence of 0.2 in 1,000 live births; (2) late-onset (LO) that presents at 7 to 89 days of life with an incidence of 0.3 to 0.4 in 1,000 live births; and (3) very-late-onset that presents after 3 months of age, mainly affects ex-extreme premature infants or immune-deficient infants. 1 2

Other rare focal infections associated with GBS infection include osteomyelitis, septic arthritis, necrotizing fasciitis, adenitis, endocarditis, cellulitis, and abscess. 2 Neonatal GBS infection is transmitted via (1) vertical transmission from a GBS colonized mother to newborn; (2) horizontal transmission from nosocomial source, community source, or less frequently after vertical GBS colonization acquired at birth. 3 Neonatal EO GBS infection is caused through the vertical transmission from a GBS colonized mother. About 10% to 50% of LO GBS infection neonates are born to mothers with positive maternal GBS screening, and the incidence tends to be higher in neonates whose mothers do not receive intrapartum antibiotic prophylaxis (IAP). 1 Neonatal colonization occurs at birth in nearly 50% of neonates born to GBS-colonized mothers who are not exposed to IAP, and these neonates will be at higher risk of both EO and LO GBS infection. 3 4 We present a case of an extreme premature neonate with a history of intracardiac migration of an umbilical venous catheter (UVC) in early life who developed GBS sepsis and sudden clinical deterioration unresponsive to aggressive medical management. The autopsy report showed multiple GBS cardiac abscesses in the myocardium in various sizes with necrotic myocardium that extended to pericardium and coronary artery; however, no abscess was found in any other organ system. In this review, we describe the plausible pathological mechanism of this rare fatal isolated multifocal GBS cardiac abscesses.

## Case Presentation

A 24.4-week GA African American extreme premature female neonate was delivered via precipitous vaginal delivery at a community hospital. The mother was a 19-year-old gravida 1 and para 1. Maternal prenatal laboratories: Human immunodeficiency virus (HIV) non-reactive, Rapid Plasma Reagin (RPR) non-reactive, *Rubella* non-reactive, Hepatitis B surface antigen (HBsAg) non-reactive, and GBS unknown. The mother did not have any significant medical history. The 24.4-week neonate's anthropometric measurements were as follows: Weight 600 g, head circumference 20.75 cm, and length 28.5 cm (Fenton growth chart ∼10%). Apgar scores were 1, 2, 5, and 6 at 1, 5, 10, and 15 minutes, respectively. The infant was intubated at birth, and a dose of surfactant was given for respiratory distress syndrome of prematurity. A UVC of size 3.5 Fr and an umbilical artery catheter (UAC) of size 3.5 Fr were placed, and she was transferred to our level 3 academic center hospital neonatal intensive care unit (NICU). Due to the risk of infection associated with extreme premature birth, empiric antibiotics (Ampicillin + Gentamicin) were given for 48 hours. Her blood culture collected at birth had remained negative for 5 days with normal white blood cell counts (WBCs), platelet counts, and C-reactive protein (CRP).

**Fig. 1:**
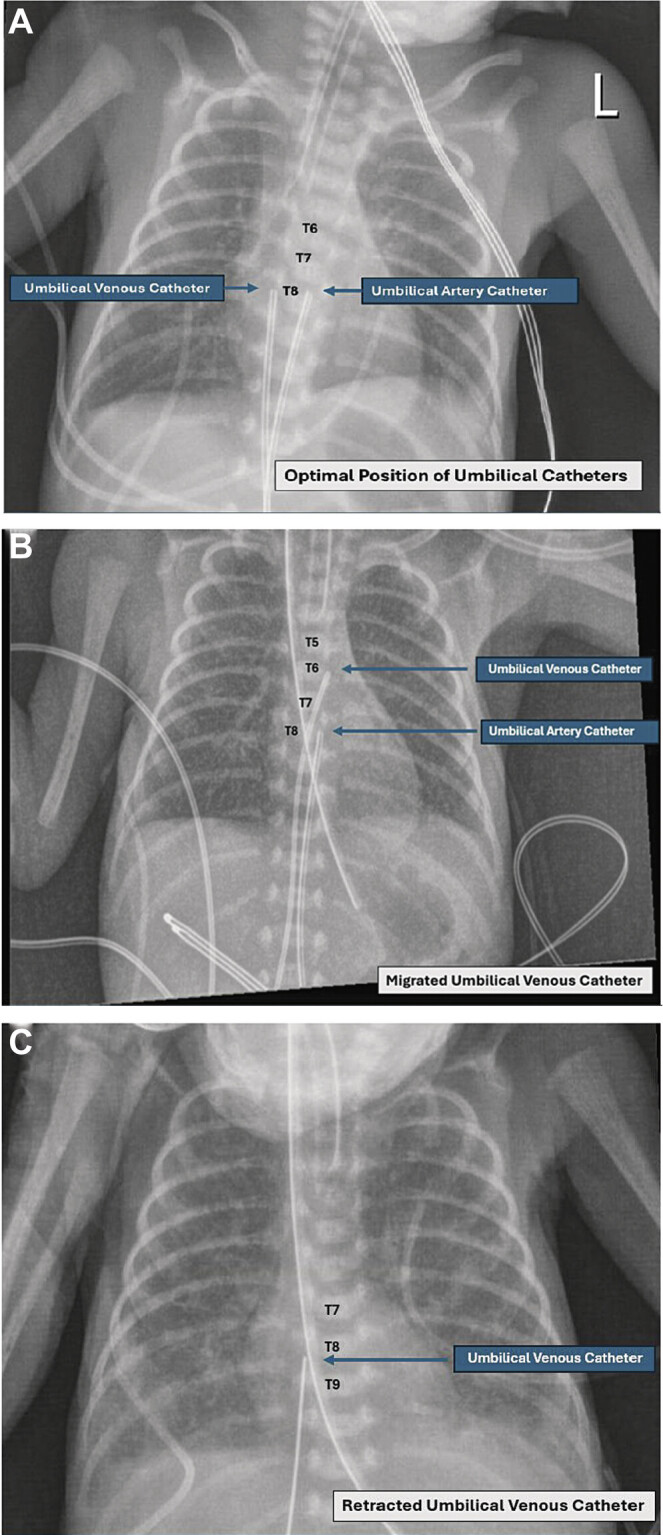
( **A** ) Anterior–posterior (AP) X-ray of optimal umbilical catheter position (on first day of life). Umbilical venous catheter (UVC) tip located at T8. Umbilical arterial catheter (UAC) tip located at T8. ( **B** ) AP X-ray of migrated UVC. UVC tip migrated to T6. UAC tip located at T8. ( **C** ) AP X-ray post-retraction of the UVC. UVC tip located at T8. UAC was removed on the fifth day of life.

**Fig. 2:**
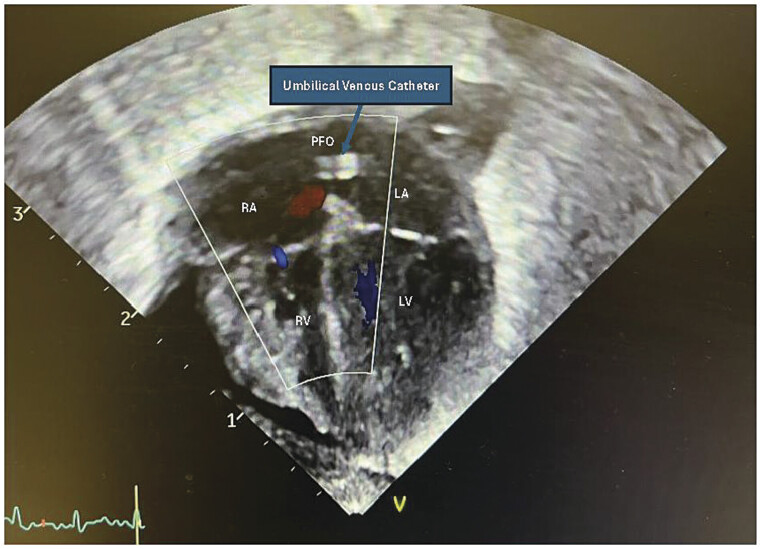
Echocardiography four-chamber view. Intracardiac migration of umbilical venous catheter (UVC) from inferior vena cava (IVC) into right atrium (RA) through patent foramen ovale (PFO) into left atrium (LA).

Post-umbilical catheter (UC) insertion, anterior–posterior (AP) thoracoabdominal (TA) X-ray confirmed both catheters were in acceptable position, with UVC tip positioned at thoracic vertebra T8 or inferior vena cava (IVC) above the diaphragm, and UAC tip positioned at thoracic vertebra T8 or descending aorta ( Fig. 1A ). Daily AP TA X-ray was obtained to ascertain the UC tip position, lungs expansion, and bowel gas pattern in the first few days of life. AP TA X-rays from the third to fifth day of life showed intermittent migration of the UVC tip to T5–T6 with suspicion that the catheter could be intermittently migrated from the IVC into the left atrium (LA) via patent foramen ovale (PFO; Fig. 1B ); the catheter position was adjusted multiple times based on the daily X-ray findings. An echocardiogram obtained to assess the status of patent ductus arteriosus (PDA) on day of life 5 showed PFO, PDA with left to right shunting, good biventricular systolic function, normal myocardium, no thrombus, no abscess, no vegetation, but the UVC tip located in LA ( Fig. 2 ). Based on both X-ray and echocardiogram findings, the UVC was retracted out again with subsequent follow-up X-rays showed ideal position of the UVC tip at T8 ( Fig. 1C ). We removed the UAC on day of life 5. While waiting for the successful placement of a percutaneously inserted central catheter (PICC) line, the UVC line remained till the time of death at the 10th day of life. Cranial ultrasound was obtained on the fourth day of life detected intraventricular hemorrhage (grade II). On day of life 10, the patient had acute clinical deterioration. Laboratory findings included severe metabolic acidosis (capillary blood gas: pH = 6.7/CO _2_ 60/HCO _3_ 7.4/BE −28), leukopenia (WBC = 3,800 μL), and elevated CRP (23 mg/dL). Empiric LO sepsis antibiotics (Vancomycin + Ceftazidime) were started. Respiratory support was escalated along with vasopressors for hypotension. Despite aggressive resuscitation efforts, the patient's clinical condition rapidly deteriorated, and the patient passed away within 6 hours of acute clinical deterioration. Blood culture grew gram-positive cocci in pairs and chains within 12 hours (bacteria specification: *Streptococcus agalactiae* or GBS; bacteria were sensitive to Vancomycin, Ceftazidime, Cefazolin, Penicillin, Ampicillin).

### Autopsy Report

**Fig. 3:**
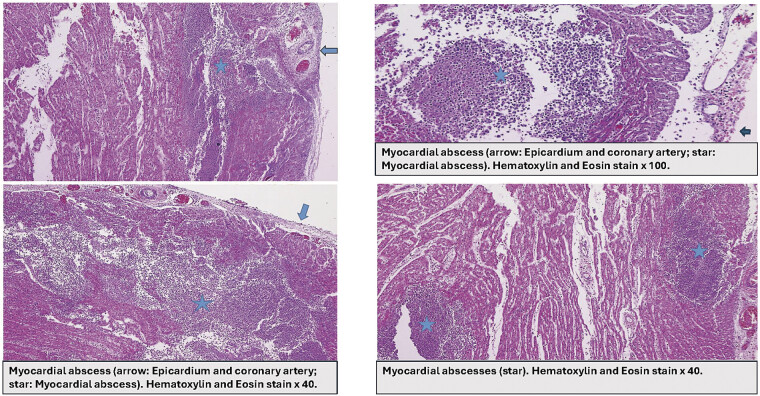
Multiple myocardial abscesses.

**Fig. 4:**
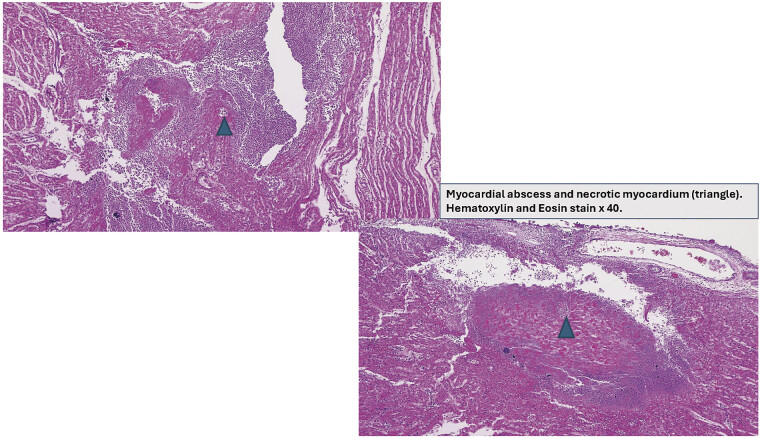
Myocardial abscesses and necrotic myocardium.

**Fig. 5:**
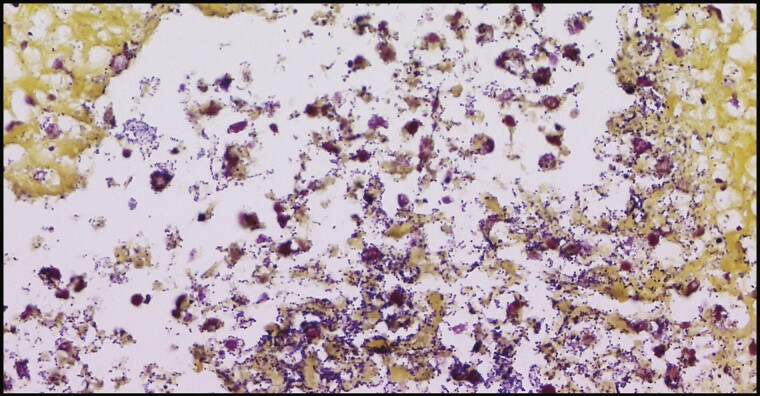
Gram-positive cocci in chains or pairs (Group B *Streptococcus* or *Streptococcus agalactiae* ). Gram stain of abscesses; ×400.

Multiple disseminated neutrophilic myocardial abscesses in variable sizes with myocardial necrosis extended to pericardium and coronary artery in anterior and posterior wall of both ventricles were identified ( Fig. 3 ). Bacterial colonization found in the necrotic myocardium ( Fig. 4 ) demonstrated gram-positive cocci in pairs and chains with gram stain, and culture was positive for GBS ( Fig. 5 ). Noticeably, no abscesses were found in any other organ system.

## Discussion

GBS sepsis with isolated cardiac abscesses, without abscesses being present in any other organ system, is a rare occurrence in neonates. In conjunction with rhythmic cardiac contractions and intracardiac blood flow dynamics, the intracardiac migrated UVC tip can cause direct trauma to the cardiac endocardium and become a potential nidus for abscess formation. In this case, the patient was born via precipitous vaginal delivery at 24.4 weeks of GA to a young-age mother with unavailable vaginorectal GBS colonization screening status. The American College of Obstetricians and Gynecologists (ACOG) recommends performing GBS colonization screening in all pregnant women at 35 to 37 weeks of pregnancy. 5 Transmission of neonatal LO GBS infection or colonization can be from a maternal or non-maternal source. In our case, we cannot assess the GBS source. It is a plausible thought that periumbilical GBS colonization in conjunction with frequent unintentional intracardiac migration of the UVC may have caused bacteria to be transferred into the bloodstream and cause GBS sepsis and myocardial abscess formation.

### Group B *Streptococcus* or *Streptococcus agalactiae*

GBS is an encapsulated β-hemolytic, catalase-negative, gram-positive cocci in pairs and/or chains bacteria. 2 The bacterial polysaccharide capsule has 10 serotypes (Ia, Ib, II, III, IV, V, VI, VII, VIII, IX). 6 In North America, serotypes Ia, Ib, II, III, and V are frequently accounted for neonatal GBS infection (98%), with serotype III being the most common cause of neonatal LO GBS and meningitis. 2 6 We did not have GBS serotyping in our case, as it was not a routine practice in our institution's laboratory. The prevalence rate of asymptomatic GBS vaginorectal intermittent or persistent colonization in pregnant women is around 10% to 30% with the incidence rate tending to be higher in lower socioeconomic conditions and younger age. 1 5 GBS has been associated with preterm birth and chorioamnionitis. 5 Intensive implementation of IAP and universal antenatal GBS screening by the ACOG 5 has drastically reduced EO GBS disease in neonates; however, the incidence of LO GBS disease remains unchanged and indeed has surpassed EO GBS disease. 2

The heterogenicity of GBS virulence factors allows the bacteria to stay in either a commensal state or an invasive state. 7 Bacterial colonization ability is mediated by GBS surface proteins, which promote adherence of the bacteria to the epithelial surface's extracellular matrix, allowing GBS to reside as a commensal bacteria in the gastrointestinal tract and/or urogenital tract. 8 Pathogenesis of GBS bacterial invasion into the epithelial barriers is via endocytosis, release of cytotoxic tissue injury toxins, and interference with host opsonization and phagocytosis capability. 7 8 9 Additionally, studies of adaptive immune response to GBS colonization in women had detected production of protective GBS serotype-specific immunoglobulin G (IgG) serum in the majority of women ≥20 years old. 10 11 In our case, the mother was ≤20 years old with a potential lack of protective GBS-specific IgG. Furthermore, routine screening of GBS colonization in neonates or colonization eradication therapy in neonates is not a current standard of practice and is not recommended. Intravenous β-lactam antibiotic targeted for GBS is the treatment of choice, which includes Penicillin G, Ampicillin, and Cefazolin. 2

### Umbilical Vascular Catheter Tip Position and Migration

Insertion of umbilical vascular catheters, whether arterial or venous, for primary central vascular access is a common practice in the NICU in the first few days of life. Placement of a UC is an important part of the management of critically ill infants in the NICU to provide parenteral nutrition, intravenous medications, frequent blood samplings, continuous central blood pressure monitoring, and transfusion of blood products. While it is an important part of NICU routine practice, the placement of UC is not a procedure without risks. Primary risks associated with UC placement are infection, thrombosis, ischemia, air emboli, hemorrhage, vascular injury or perforation, vasospasm, extravasation, internal organ injury or perforation, catheter occlusion, catheter leakage, catheter breakage, catheter malposition, and catheter migration. 12 13 14 15

### Umbilical Arterial Catheter

The preferred optimal UAC tip position is in the aorta away from any major organ-feeding vessels, with “high-line” UAC tip located at thoracic vertebra T6–T9 ( Fig. 1A ) and “low-line” UAC tip located at lumbar vertebra L3–L5 on AP TA X-ray. 14 16 17 Malposition or migration of UAC is very rare, but it can happen, causing serious life-threatening complications such as bowel perforation, necrotizing enterocolitis, aortic dissection, aortic aneurysm, aortic rupture, bladder perforation, renal artery thrombosis-associated hypertension, lower extremities ischemia, spinal cord infarction, paraplegia, and loss of limb. 12 17 Another frequent UAC complication is transient arterial vasospasm and intra-arterial thrombotic ischemia with discoloration or gangrene of the toes, buttocks, and spine, which are more often seen with “low-line” UAC tip position. 18

### Umbilical Venous Catheter

The preferred optimal UVC tip position is in the IVC above the diaphragm and outside the heart or in the vena cava–right atrial junction, which corresponds to the thoracic vertebrae T8–T10 landmark on AP TA X-ray or slightly (∼1 cm) above the diaphragm on lateral TA X-ray ( Fig. 1A, C ). 13 14 16 17 The migration of UVC can cause life-threatening complications, such as pericardial effusion, pericardial hematoma, cardiac tamponade, cardiac arrhythmia, hydrothorax, liver hematoma, liver necrosis, liver abscess, peritoneal effusion, necrotizing enterocolitis, and bowel perforation. 15 19 20 Malposition or migration of UVC occurs more frequently than UAC, with a reported incidence of approximately 50% to 90%. 21 22 23 UVC migration tends to occur in the first 24 to 48 hours of catheter placement, with a higher incidence tendency seen in extreme premature or extreme low birth weight neonates. 24 This is particularly due to certain neonatal conditions can cause inward or outward migration of the UVC. Inward migration of the UVC can be due to drying and shriveling of the umbilical cord's Wharton jelly or umbilical stump, increased lung volume expansion, increased invasive respiratory support, increased work of breathing, tachycardia, decreased abdominal girth, flexed knee position, using a large-sized catheter, or frequent repositioning of the catheter. 25 26 27 Outward migration of UVC can be caused by the opposite conditions.

Correct technique of securing the UC position after placement, and daily catheter position assessment, is crucial to avoid migration of the catheter. 28 In our center, the UC is secured using a suture to the umbilical stump after confirmation of the ideal position by AP TA X-ray, and then the external part of the catheter is secured to the abdominal skin using Tegaderm®. Currently, there is no literature to strongly support which technique of securing the UC is superior; henceforth, each center should select its own protocol and periodically review their practice quality. 29 30 The most commonly used and rapidly available method of confirming UC tip position is by AP TA X-ray or bedside point-of-care ultrasound, depending on the available resources. 31 Moreover, other highly operator-dependent methods, such as echocardiography and ultrasonography, can also be used. 13 32 33

### Central Line-Associated Bloodstream Infection

Central line-associated bloodstream infection is one of the crucial infection concerns in the NICU. 34 The Centers for Disease Control and Prevention (CDC) recommends removing any central line access, including UC, as soon as possible when no longer needed for clinical care. 35 Infection risk increases when UC is in place for >7 days and is significantly higher when the UC dwelling duration surpasses >14 days. 36 37 38 The current CDC conditional recommendation is to consider removal of UC at or before 7 days. 35 If UVC is still required for clinical care, the CDC recommends considering transition to PICC line at ≤7 days whenever the clinical condition allows. Similar to UC (UVC or UAC), the PICC line is also considered a central catheter access. Therefore, using the PICC line as a central line access option is also not without risks of infection or other catheter-associated complications. 34 36 38 Aseptic central line insertion and care bundles, together with implementation of quality improvement initiatives, are part of the essential practice to prevent central line infection in the NICU. 35 36 38 These strategies have been shown to significantly reduce the rate of central line infection or complication in NICU. 38 Moreover, the CDC recommends low-dose heparin infusion (0.25–1 unit heparin/mL) to prevent UC occlusion-associated infection and embolic event, but topical antimicrobial application is not recommended to prevent UC-associated infection. 34

### Cardiac Abscess

Cardiac abscess is a rare, life-threatening suppurative infection of the endocardium or myocardium. Infective agent can reach the heart through (1) systemic bacteremia, (2) infective endocarditis, (3) direct trauma or injury, (4) direct extension of the preexisting infective focus, and (5) intracardiac device (stent, occluder, pacemaker, prosthetic valve, cardiac indwelling catheter, etc.). 39 In our case, intracardiac UVC tip migration caused direct injury to the cardiac tissue. Frequent adjustment of the UVC could also cause bacteria to be transferred from the colonized umbilical site into the bloodstream, resulting in bacterial proliferation into the injured cardiac tissue. Myocardial abscess can damage cardiac muscle; this will lead to cardiac muscle contractility dysfunction and heart failure. Cardiac abscess can also damage Purkinje fibers, causing cardiac conduction dysfunction and sudden cardiac arrest. Cardiac abscesses can occlude coronary arteries, resulting in myocardial infarction. These could have been the contributing factors for the sudden clinical decompensation in our patient.

Although GBS is not the most common bacteria causing abscess formation, the heterogenous pathogenic virulence factors released by GBS bacteria can cause the formation of colonization, cellular invasion, and the formation of abscesses. Detection of cardiac abscess can be done using echocardiogram, cardiac magnetic resonance imaging (MRI), or cardiac computed tomography (CT). Nevertheless, the majority of cardiac abscess cases were discovered during postmortem autopsy. 40 In our case, cardiac abscesses were detected on autopsy, with histopathology results showing myocardial necrosis with various sizes of neutrophilic myocardial abscesses and GBS colonization ( Figs. 3 and 4 ). As in adults or older children, cardiac abscess in extreme premature neonates is rare and fatal. Antibiotic therapy, supportive therapy, and surgical intervention are the management of choice; however, that may not always be successful.

## Conclusion

Intracardiac migration of UVC can cause endocardial and/or myocardial damage. Inward migration of UC can also transfer bacteria that are colonized in the periumbilical area into the bloodstream, causing bacteremia and sepsis. With GBS sepsis, the injured cardiac tissue became a nidus for multiple myocardial abscesses and fatal cardiac dysfunction. Our patient died from fulminant GBS sepsis along with isolated multiple cardiac abscesses in the myocardium. Therefore, it is prudent to use the correct aseptic technique of securing and managing the UC (UVC) as well as daily assessment of the UC (UVC) position.

## References

[1] Coggins S A Puopolo K M Neonatal Group B *Streptococcus* disease Pediatr Rev 2024 45 02 63 73 38296778 10.1542/pir.2023-006154 PMC10919294

[2] Committee on Infectious Diseases American Academy of Pediatrics Group B streptococcal infections 2024

[3] Miselli F Frabboni I Di Martino M Transmission of Group B *Streptococcus* in late-onset neonatal disease: A narrative review of current evidence Ther Adv Infect Dis 2022 9 20499361221142732 10.1177/20499361221142732 PMC9780763 36569815

[4] Berardi A Trevisani V Di Caprio A Understanding factors in group B *Streptococcus* late-onset disease Infect Drug Resist 2021 14 3207 3218 34429620 10.2147/IDR.S291511 PMC8380284

[5] American College of Obstetricians and Gynecologists Committee Opinion No. 797: Prevention of group B streptococcal early-onset disease in newborns: Correction Obstet Gynecol 2020 135 04 978 979 10.1097/AOG.0000000000003824 32217968

[6] Raabe V N Shane A L Group B *Streptococcus* ( *Streptococcus agalactiae* ) Microbiol Spectr2019 7(02):10.1128/microbiolspec.gpp3-0007-2018 10.1128/microbiolspec.gpp3-0007-2018 PMC6432937 30900541

[7] Rajagopal L Understanding the regulation of group B streptococcal virulence factors Future Microbiol 2009 4 02 201 221 19257847 10.2217/17460913.4.2.201 PMC2691590

[8] Doran K S Nizet V Molecular pathogenesis of neonatal group B streptococcal infection: No longer in its infancy Mol Microbiol 2004 54 01 23 31 15458402 10.1111/j.1365-2958.2004.04266.x

[9] Burnham C AD Tyrrell G J Virulence factors of group B streptococci Rev Res Med Microbiol 2003 14 04 109 118

[10] Campbell J R Hillier S L Krohn M A Ferrieri P Zaleznik D F Baker C J Group B streptococcal colonization and serotype-specific immunity in pregnant women at delivery Obstet Gynecol 2000 96 04 498 503 11004347 10.1016/s0029-7844(00)00977-7

[11] Baker C J Carey V J Rench M A Maternal antibody at delivery protects neonates from early onset group B streptococcal disease J Infect Dis 2014 209 05 781 788 24133184 10.1093/infdis/jit549 PMC3923540

[12] Gibson K Sharp R Ullman A Morris S Kleidon T Esterman A Adverse events associated with umbilical catheters: A systematic review and meta-analysis J Perinatol 2021 41 10 2505 2512 34272469 10.1038/s41372-021-01147-x

[13] Greenberg M Movahed H Peterson B Bejar R Placement of umbilical venous catheters with use of bedside real-time ultrasonography J Pediatr 1995 126 04 633 635 7699547 10.1016/s0022-3476(95)70366-7

[14] Michel F Brevaut-Malaty V Pasquali R Comparison of ultrasound and X-ray in determining the position of umbilical venous catheters Resuscitation 2012 83 06 705 709 22155219 10.1016/j.resuscitation.2011.11.026

[15] Oestreich A E Umbilical vein catheterization–appropriate and inappropriate placement Pediatr Radiol 2010 40 12 1941 1949 20890597 10.1007/s00247-010-1840-2

[16] Anderson J Leonard D Braner D A Lai S Tegtmeyer K Videos in clinical medicine. Umbilical vascular catheterization N Engl J Med 2008 359 15 e18 18843120 10.1056/NEJMvcm0800666

[17] Liszewski M C Daltro P Lee E Y Back to fundamentals: Radiographic evaluation of thoracic lines and tubes in children AJR Am J Roentgenol 2019 212 05 988 996 30779658 10.2214/AJR.18.20704

[18] Barrington K J Umbilical artery catheters in the newborn: Effects of position of the catheter tip Cochrane Database Syst Rev 2000 1999 02 CD000505 10796375 10.1002/14651858.CD000505 PMC7038652

[19] Henderson R DE Padash S Adams S J Augusta C Yi X Babyn P Neonatal catheter and tube placement and radiographic assessment statistics in relation to important anatomic landmarks Am J Perinatol 2024 41(S 01):e2299 e2306 37494483 10.1055/s-0043-1771051

[20] Schlesinger A E Braverman R M DiPietro M A Pictorial essay. Neonates and umbilical venous catheters: normal appearance, anomalous positions, complications, and potential aid to diagnosis AJR Am J Roentgenol 2003 180 04 1147 1153 12646473 10.2214/ajr.180.4.1801147

[21] Dubbink-Verheij G H Visser R Tan R NGB Roest A AW Lopriore E Te Pas A B Inadvertent migration of umbilical venous catheters often leads to malposition Neonatology 2019 115 03 205 210 30645997 10.1159/000494369 PMC6518856

[22] Hoellering A Tshamala D Davies M W Study of movement of umbilical venous catheters over time J Paediatr Child Health 2018 54 12 1329 1335 29806878 10.1111/jpc.14073

[23] Plooij-Lusthusz A M van Vreeswijk N van Stuijvenberg M Bos A F Kooi E MW Migration of umbilical venous catheters Am J Perinatol 2019 36 13 1377 1381 30620943 10.1055/s-0038-1677016

[24] Harabor A Soraisham A Rates of intracardiac umbilical venous catheter placement in neonates J Ultrasound Med 2014 33 09 1557 1561 25154935 10.7863/ultra.33.9.1557

[25] D'Andrea V Prontera G Rubortone S A Umbilical venous catheter update: A narrative review including ultrasound and training Front Pediatr 2022 9 774705 35174113 10.3389/fped.2021.774705 PMC8841780

[26] Fleming S E Kim J H Ultrasound-guided umbilical catheter insertion in neonates J Perinatol 2011 31 05 344 349 21311495 10.1038/jp.2010.128

[27] Soonsawad S Kieran E A Ting J Y AlonsoPrieto E Panczuk J K Factors associated with umbilical venous catheter malposition in newborns: A tertiary center experience Am J Perinatol 2022 39 16 1805 1811 33853146 10.1055/s-0041-1726385

[28] Elser H E Options for securing umbilical catheters Adv Neonatal Care 2013 13 06 426 429 24300962 10.1097/ANC.0000000000000038

[29] D'Andrea V Prontera G Pinna G Securement of umbilical venous catheter using cyanoacrylate glue: A randomized controlled trial J Pediatr 2023 260 113517 37244573 10.1016/j.jpeds.2023.113517

[30] Perl J R Crabtree-Beach T Olyaei A Hedges M Jordan B K Scottoline B Reducing umbilical catheter migration rates by using a novel securement device J Perinatol 2024 44 09 1359 1364 38521880 10.1038/s41372-024-01943-1 PMC11379623

[31] D'Andrea V Prontera G Cota F Real-time ultrasound tip location reduces malposition and radiation exposure during umbilical venous catheter placement in neonates: A retrospective, observational study Neonatology 2025 122 01 32 37 10.1159/000538905 38934171

[32] Ades A Sable C Cummings S Cross R Markle B Martin G Echocardiographic evaluation of umbilical venous catheter placement J Perinatol 2003 23 01 24 28 12556923 10.1038/sj.jp.7210851

[33] Franta J Harabor A Soraisham A S Ultrasound assessment of umbilical venous catheter migration in preterm infants: A prospective study Arch Dis Child Fetal Neonatal Ed 2017 102 03 F251 F255 28424358 10.1136/archdischild-2016-311202

[34] Healthcare Infection Control Practices Advisory Committee (HICPAC) (Appendix 1) O'Grady N P Alexander M Burns L A Summary of recommendations: Guidelines for the Prevention of Intravascular Catheter-related Infections Clin Infect Dis 2011 52 09 1087 1099 21467014 10.1093/cid/cir138 PMC3106267

[35] Centers for Disease Control and Prevention. Recommendations for Prevention and Control of Infections in Neonatal Intensive Care Unit Patients: Central Line-associated Blood Stream Infections Guideline. 2022.

[36] Butler-O'Hara M Buzzard C J Reubens L McDermott M P DiGrazio W D'Angio C T A randomized trial comparing long-term and short-term use of umbilical venous catheters in premature infants with birth weights of less than 1251 grams Pediatrics 2006 118 01 e25 e35 16785289 10.1542/peds.2005-1880

[37] Levit O L Shabanova V Bizzarro M J Umbilical catheter-associated complications in a level IV neonatal intensive care unit J Perinatol 2020 40 04 573 580 31911645 10.1038/s41372-019-0579-3

[38] Centers for Disease Control and Prevention. Recommendations for Prevention and Control of Infections in Neonatal Intensive Care Unit Patients: Central Line-associated Blood Stream Infections Appendix. 2022

[39] Ramos Tuarez F J Yelamanchili V S Sharma S Law M A Cardiac Abscess StatPearls 2025 29083576

[40] Kim H-S Weilbaecher D G Lie J T Titus J L Myocardial abscesses Am J Clin Pathol 1978 70 01 18 23 696668 10.1093/ajcp/70.1.18

